# Altered Pattern of Immunoglobulin A-Targeted Microbiota in Inflammatory Bowel Disease After Fecal Transplantation

**DOI:** 10.3389/fmicb.2022.873018

**Published:** 2022-06-22

**Authors:** Wen-qi Huang, Hong-Li Huang, Wu Peng, Yan-Di Liu, You-Lian Zhou, Hao-Ming Xu, Liang-jie Zhang, Chong Zhao, Yu-Qiang Nie

**Affiliations:** ^1^Department of Gastroenterology, School of Medicine, The Second Affiliated Hospital, South China University of Technology, Guangzhou, China; ^2^Department of Gastroenterology, Guangzhou Digestive Disease Center, Guangzhou First People’s Hospital, Guangzhou, China; ^3^Department of Infectious Disease, The First Affiliated Hospital of Bengbu Medical College, Bengbu, China

**Keywords:** inflammatory bowel disease, immunoglobulin A, immunoglobulin G, gut bacteria, fecal microbiota transplant

## Abstract

Adaptive immune response to the gut microbiota is one of the main drivers of inflammatory bowel disease (IBD). Under inflammatory conditions, immunoglobulin (Ig)-targeted bacteria are altered. However, changes in Ig-targeted bacteria in Asian patients with IBD with ulcerative colitis (UC) remain unclear. Furthermore, changes in IgA-targeted bacteria in patients with UC treated with fecal microbiota transplantation (FMT) are unclear. Here, we analyzed fecal samples of patients with IBD and patients with UC before and after FMT by flow cytometry. We found that the percentage of IgA/G-coated bacteria can be used to assess the severity of IBD. Besides oral pharyngeal bacteria such as *Streptococcus*, we hypothesized that *Megamonas*, *Acinetobacter*, and, especially, *Staphylococcus* might play an important role in IBD pathogenesis. Moreover, we evaluated the influence of FMT on IgA-coated bacteria in patients with UC. We found that IgA-bacterial interactions were re-established in human FMT recipients and resembled those in the healthy fecal donors. Additionally, the IgA targeting was not influenced by delivery methods: gastroscopy spraying and colonic transendoscopic enteral tubing (TET). Then, we established an acute dextran sulfate sodium (DSS)-induced mouse model to explore whether FMT intervention would impact IgA/G memory B cell in the intestine. We found that after FMT, both IgA/G memory B cell and the percentage of IgA/G-targeted bacteria were restored to normal levels in DSS mice.

## Introduction

Intestinal mucosal inflammatory damage is one of the characteristics of the pathogenesis in inflammatory bowel disease (IBD). This inflammatory damage can be triggered by innate and adaptive immune responses (Sun et al., 2015; Sartor and Wu, 2017). Additionally, the microbiota plays an essential role in many diseases, especially in the development of IBD and other immune diseases (Sansonetti, 2004). However, the precise understanding of immune interactions with the microbiota in IBD remains unclear (Qin et al., 2010). Immunoglobulin A (IgA) is the most abundant antibody secreted into the gastrointestinal tract and provides the first line of immune protection, while IgG is the most abundant antibodies in peripheral blood (Macpherson et al., 1996). Secretion of IgA is induced by both food antigens and the intestinal microbiota (Bunker Jeffrey et al., 2017). Furthermore, Bendelac etc. in ‘Natural polyreactive IgA antibodies coat the intestinal microbiota’, suggested ‘endogenous mechanism driving homeostatic production of polyreactive IgAs with innate specificity to microbiota’, which also indicates the relationship between IgA and gut microbiota (Hapfelmeier et al., 2010).

Secretion of IgA helps regulate the microbiota, altering gene expression patterns and adherence to tissue-cultured intestinal epithelial cells (Bollinger et al., 2003; Suzuki et al., 2004; Peterson et al., 2007; Mathias et al., 2010). Several studies that combined flow cytometry sorting with 16S rDNA sequencing found that IgA and IgG can bind colitogenic members of the microbiota, and that IgA limits the immune response of T helper (Th) cells to mucosal commensal antigens (Palm et al., 2014; Bunker et al., 2015; Koch et al., 2016). Active patients with IBD have been reported to have higher percentage of IgA/G-coated microbiota than healthy controls (van der Waaij et al., 2004; Peterson et al., 2007). Therefore, evaluating the IgA/G-coated microbiota might be important in the treatment of IBD (Rengarajan et al., 2020). Fecal microbial transplantation (FMT) involves the administration of a healthy fecal material containing approximately 10^11^ bacterial cells/g, as well as viruses, fungi, and archaea, into the intestinal tract of a recipient (Bojanova and Bordenstein, 2016). FMT is recognized as a successful therapy for *Clostridioides difficile* infections and has also been explored as a promising therapy for IBD compared to antibiotics or probiotics (Weingarden and Vaughn, 2017). Recent studies have shown that IgA-bacterial interactions can be re-established by FMT and resemble the healthy fecal donor in patients with recurrent *Clostridioidesdifficile* infection (Huus et al., 2021).

However, several questions remain unanswered regarding IgA/G-coated microbiota in patients with IBD patients FMT therapy. First, IBD is a chronic remittent inflammatory disease, and the relationship among IgA/G-targeting bacteria, disease activity, and clinical characteristics in Asian patients with IBD has not been fully explored. Second, the altered pattern of IgA/G targeted bacteria in IBD following FMT as well as its underlying mechanism have not been reported. Moreover, it remains unclear whether IgA/G production after FMT in patients with IBD increases through IgA/G + B cells.

Therefore, in this study, we characterized the IgA/G-coated bacteria in 61 patients with IBD, including 18 before and after FMT, by fluorescence-activated cell sorting (FACS) combined with 16S rDNA sequencing. We also demonstrated the influence of different delivery methods, including colonic TET and gastroscopy spraying, and analyzed IgA-targeting bacteria donor and host pre/post-transplant in IBD FMT recipients. A previous report found that IgA-coated *Streptococcus* was a dominant biomarker in a CD-based IBD cohort in the United States. Herein, we found a higher percentage of Ig-coated *Megamonas* and *Acinetobacter* in patients with IBD and higher IgA-coated *Staphylococcus aureus* in patients with CD. Consistent with the previous study, Ig-bound bacteria might be used as biomarkers in the assessment of IBD activity. Furthermore, we evaluated the influence of FMT on IgA-coated bacteria in the patients with UC. We found that healthy IgA–microbiota interactions were restored in the patients with UC after FMT. Moreover, the delivery method did not influence IgA targeting. The patterns of IgA-coated bacteria in FMT recipients closely resembled those in the donors. Finally, we established an acute DSS-induced mouse model to explore whether FMT intervention would impact IgA/G + B cells and showed that it decreased the production and percentage of IgA/G-bound bacteria.

## Materials and Methods

### Patients and Volunteers

The study collected samples of patients with IBD from the Guangzhou First People’s Hospital from June 2020 to October 2021. In addition to clinical symptoms, clinical laboratory indicators and imaging tests, the patients with IBD were diagnosed by histological examination of the ileum and colon biopsy. Prior to inclusion in the study, written informed consent was obtained from all the study participants. The patients’ other disease activity index (CDAI; part of Mayo score) and laboratory test results were collected within 5 days of collecting stool samples. All the patients with IBD met the standard of not having not received antibiotics and probiotics for at least 3 months. We collected stool samples within 1 week before FMT and 4 weeks after FMT, and stored them at −80°C for IgA/G-seq as previously reported. Healthy people that match the age and sex of the patients with IBD consist the group that was compared with the IBD cohort. In addition, samples of donors for each FMT operation are also collected. There are mainly two donors who provided the source of bacteria for FMT therapy. We excluded subjects with any other gastrointestinal diagnosis or who had bowel resection. There was no statistical difference in age, gender, and BMI of the patients in the cohort. Whole samples were analyzed with a mixed-effects regression model and corrected for the random effects of each subject.

### Intervention by Fecal Microbiota Transplantation

Fecal samples from the healthy donors (approximately 150–200 g each) were dissolved in 1,000 ml of normal saline, and the microbiota was isolated using standard protocols recommended by the manufacturer [performed using a GenFMTer automated purification system (FMT Medical)]. Fresh feces from currently healthy male student donors were used for each fecal transplant, and the cohort used feces from a total of two different donors. Nine patients with UC received stool from s #3, and 6 patients with UC received stool from donor #8. The remaining 3 patients with UC who received feces from other different donors were not included in the discussion. There are two main ways of fecal bacterial transplantation in this study: TET tube placement under colonoscopy and spraying under gastroscope. Eighteen patients were randomly selected for surgery with enteroscopic TET tube or gastroscopic spraying, 8 patients underwent enteroscopic TET tube placement, and the other 10 patients underwent gastroscopic spraying for surgery. Colonoscopy with TET tube: 1 day after TET tube placement, 150 ml of normal saline containing the microbiota (∼50 cm^3^) was injected into the colon through a TET catheter *via* a 50-ml syringe. After FMT, the patients were kept in the right side position for ≥ 30 min and allowed to eat after 2 h. FMT was repeated every other day for a total of 3 times of treatment. Gastric spraying: after fasting for more than 8 h, the patients underwent sedative gastroscopy surgery and sprayed on the upper duodenum. The contents, volume, and treatment times were the same as above. After FMT, the patients remained in a semi-recumbent position for ≥ 30 min, and 2 h later were allowed to eat.

### 16S rDNA Analysis

A sorted bacterial suspension was boiled at 100°C for 15 min, and then 2 μl of lysate was used as a template for 16S PCR, and previously described barcode primers were used to amplify the bacterial V4 hypervariable region of 16S rDNA. PCR was performed under the following cycling conditions using Phusion Polymerase: initial denaturation at 98°C for 5 min, 98°C for 20 s, 55°C for 15 s, 72°C for 30 s, and final extension for 10 min, a total of 30 cycles at 72°C. The reaction was performed on gel to ensure successful amplification, and purified and standardized using a 96-well Sequel-Prep kit (Thermo Fisher Scientific A1051001). All reactions were then combined and gel-extracted (GeneJet K0692) to remove primer dimers. The combined PCR products were sequenced using an Illumina MiSeq platform (2 × 250-bp double-ended reads) (Kozich et al., 2013).

### Bioinformatics Analysis of 16S rDNA Data

The sequences were then analyzed in QIIME21 (Bolyen et al., 2019), in which we quality controlled the sequences with Dada2, and then trimmed to 250 bp. Quality tags were clustered into operational taxonomic units (OTUs) of ≥ 97% similarity through UPARSE, and each cluster was the most abundant tag sequence (Edgar, 2013). The analysis of flower/Venn figures and PCoA were operated through the flower/Venn diagram package and unifrac distance, respectively (Caporaso et al., 2010; Chen and Boutros, 2011). Furthermore, diversity indexes were analyzed with the QIIME software. We used the ggplot2 package (version 2.2.1) to draw the OTU rarefaction and rank abundance curves. We used the Welch’s *t*-test or Wilcoxon rank test to analyze differences between two groups and used Tukey’s HSD test or Kruskal–Wallis *H*-test to analyze differences between three or more groups in the R project Vegan package (version 2.5.3). Furthermore, the biomarker features in each group were screened using the labdsv package (version 2.0.1) in the R project. Lastly, we extracted the 16S rRNA gene sequence of the prokaryotic whole genome from the KEGG database and compared it to the SILVA SSU Ref NR database (BLAST bit score > 1,500) using the BLASTN algorithm to establish a correlation matrix, and performed Tax4Fun function prediction on the bacteria bound by IgA.

### Bacteria Immunoglobulin A/Immunoglobulin G-Seq

Fecal IgA- and IgG-coated bacteria was designed by flow cytometry as reported previously (Palm et al., 2014; Huus et al., 2020, 2021). A 50-mg chip of stool was obtained from each fecal sample and stored at −80°C. The sample was put into Fast Prep Lysing Matrix D tubes containing ceramic beads (MP Biomedicals) and homogenized in 1 ml phosphate-buffered saline (PBS; GBICO, C10010500BT) by bead beating (Mini-Beadbeater; BioSpec). Then, fecal pellets were centrifuged gently to separate large particles. A volume of 100-μl supernatants were collected from the sample and washed thrice with the staining buffer (PBS containing 1% bovine serum albumin). The samples were then blocked for 20 min with 20% normal mouse serum for human samples or 20% normal rat serum for mouse samples (Jackson ImmunoResearch). After blocking, the samples were stained with anti-human IgA/G-PE (Miltenyl 130–093-128) or anti-mouse IgA-apc/anti-mouse IgG-PE (biolegend) at 1:25 dilution for 30 min in the dark. After being washed thrice, the samples were analyzed by flow cytometry (Sony ID7000) to decrease the autofluorescence from bacteria and set the gate of FSC and SSC. After the setting, the samples were sorted into IgA-positive bacteria and IgA-negative bacteria by flow cytometry (Sony SH800S). Each of the sorted samples have collected a minimum of 800,000 events and frozen at −20°C for further 16S analysis.

### Fecal and Serum Immunoglobulin A/Immunoglobulin G Quantification by ELISA

The measurements of soluble IgA and IgG were quantified by enzyme-linked immunosorbent assay (ELISA; Cloud clone, SEA546Hu/CEA544Hu). After freeze drying, the fecal samples were weighted approximately 10 mg and then ultrasonicated in 2 ml PBS containing EDTA, soybean trypsin inhibitor (Gibco). The samples were centrifuged at 1,800 rpm and 4°C for 10 min. After another centrifugation at 14,000 rpm and 4°C for 15 min, the supernatants were collected and stored at −80°C. The ELISA plates were coated with anti-human IgA or IgG. The experimental samples and standards were added to the wells and incubated at 37°C for 1 h. After washing with an ELISA washer, anti-IgA or IgG with HRP-conjugated streptavidin was added for 1 h. Lastly, the TMB was added to the wells in the dark, and the results were analyzed with a VICTOR Nivo multimode plate reader (PerkinElmer).

### Establishment of Dextran Sulfate Sodium-Induced Colitis Mouse Model

In this study, the mouse model of acute colitis induced by DSS was used. Male BALB/c mice aged 6–8 weeks were purchased from Guangdong Medical Experimental Animal Center (GDMLAC; certificate number SYXK 2018-0002, Foshan, China). The mice were kept in a room free of specific pathogens, with a 12-h light/dark cycle, constant temperature (24°C) and humidity (50–70%), and free access to food and water. The experimental procedure was approved by the Animal Ethics Committee of Guangzhou First People’s Hospital (acceptance no: 2017-202).

Mice with acute colitis were randomly divided into three groups: the control group, the DSS enteritis model group, and the human fecal FMT group. The control group was free to drink water, and the model group and intervention group were free to drink 3% DSS for 7 days. The control group (*n* = 6) and the model group (*n* = 6) were given phosphate buffered saline (PBS, 0.1 ml/10 g) by gavage. The human fecal FMT group was fed with fresh fecal bacteria (0.1 ml/10 g) prepared from the healthy donors. The weight of the mice, consistency of feces, and whether there is blood in the anus or feces are tested every day. In addition, according to a modified version of as previously described method (Murthy et al., 1993), the disease activity index (DAI) of a single mouse needs to be scored every day, and at the end of the experiment, their blood and stool samples and colon tissue were collected. The length of the colon was measured, and the dissected tissue was fixed overnight in 4% paraformaldehyde or fixed with CARNOY’s Fixative at 4 degrees for no more than 2 h before dehydration and embedding.

### Hematoxylin and Eosin, Immunohistochemistry, and Immunofluorescence to Evaluate the Barrier Function of the Intestinal Tract

The mice were euthanized, and the entire colon tissue from the cecum to the anus was dissected. We gently removed the intestinal contents and flushed the intestinal cavity with saline. The tissues were fixed by 10% buffered formalin paraffin or carnoy fixative. They were then dehydrated by 100% absolute ethanol 15 min*2 times and xylene 15 min*2 times before soaking in wax. Tissue sections (5 μm) were stained with hematoxylin and eosin (H&E). The HE sections were blindly scored by an experienced pathologist. Histological scoring was performed using a modified scoring system reported previously (Iqbal et al., 2002). In brief, longitudinal sections were examined as followed: (A) the degrees of inflammation (0: no inflammatory infiltrate, 1: infiltrates in the lamina propria, 2: infiltrates in the submucosa, and 3: transmural infiltration), (B) ulceration (0: no ulceration, 1: one or two ulcers, 2: three or four ulcers, and 3: more than four ulcers), (C) mucosal hyperplasia (0: normal, 1: slightly thickened mucosa with minimal fibrosis, 2: mucosal thickening with fibrous hyperplasia, and 3: extensive mucosal thickening and fibrous hyperplasia or granulation), and (D) edema (0: none, 1: 0–30%, 2: 30–70%, and 3: > 70%). Whole lesion severity score was estimated by summation of the products of extent and intensity scores for every individual lesion component.

After deparaffinization and hydration, the sections were repaired with citrate antigen, and the repaired sections were restored with 3% H_2_O_2_ to expose the antigenic sites, and incubated with anti-ZO-1 (ab216880) from Abcam (Cambridge, MA, United States) (ab168986) or anti-muc2 (272692) overnight at 4°C. We used DAB to develop occludin. ZO1 and muc2 used fluorescent secondary antibodies with 555 and 488 fluorescein, respectively. To visualize bacterial and cellular DNA, 26 slides were counterstained with 4’,6-diamidino-2-phenylindole (DAPI, 1 min).

### Mouse Intestinal Tissue Flow Cytometry

The flow cytometric antibodies used in mouse intestinal tissues are: anti-CD45, anti-CD19, anti-IgA, and anti-IgG antibodies, purchased from Biolegends (San Diego, CA), and the method of flow sorting is as previously described (Cao et al., 2012). The mouse intestinal tissues were seperated into single cell suspension. They were then washed with PBS before incubating the cells with various conjugated IgA/IgG antibody. After fixing in 1% buffered paraformaldehyde, the single cell suspension was then quantified through flow cytometer (BD Bioscience). We used FlowJo for data analysis. We used mAb with the same isotype but unrelated specificity as a negative control.

### Statistical Analysis

The statistical analysis of this study was carried out with the phyloseq and ggplot2 packages in R studio, shown in Supplementary Material for details. We conducted a principal component analysis based on Euclidean distance to explore the data structure of the IgA index. PERMANOVA analysis iteration was conducted on each variable of interest to determine its contribution to the exponential distribution of IgA. The data were expressed as a percentage or mean ± standard deviation (SD). Differences between groups or were analyzed by paired *t*-test, unpaired *t*-test, χ^2^-test, or Wilcoxon signed rank test, as well as one-way analysis of variance and *post hoc* Tukey’s test (if applicable). Spearman correlation was applied to continuous variables, and a *p*-value of < 0.05 indicated a statistically significant difference.

## Results

### Higher Immunoglobulin A/Immunoglobulin G-Targeting Microbiota Was Detected in Patients With Inflammatory Bowel Disease

**FIGURE 1:**
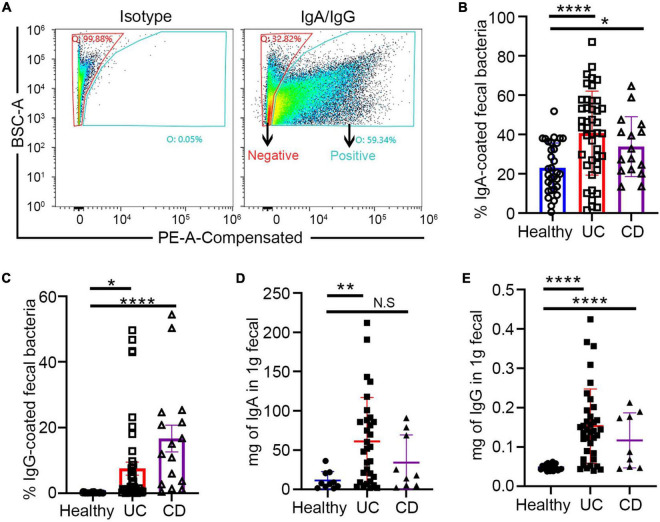
Increased fecal free Ig and Ig-bound bacteria in patients with inflammatory bowel disease (IBD). **(A)** Fluorescence-activated cell sorting (FACS) profiles of Ig-bound gut bacteria. **(B,C)** Increased Ig-bound fecal bacteria in patients with IBD. **(D,E)** Free fecal Ig by ELISA in patients with IBD and healthy controls. *p < 0.05, **p < 0.01, ***p < 0.001, ns means no significance difference.

In this study, fecal and blood samples were obtained from 61 randomly selected patients with IBD from Guangzhou First People’s Hospital. Among them, 18 patients whose basic treatment was mesalamine came for FMT treatment because of recurrence of symptoms and increased disease activity score. The details of FMT intervention has been described above. The fecal and blood samples were collected from them before and after treatment (Table 1). The fecal samples were sorted into IgA/G + and IgA/G- coated bacteria by flow cytometry and identified by 16S rDNA sequencing (Figure 1A). We found that the patients with IBD presented a higher percentage of IgA/G-coated bacteria than the healthy controls, consistent with previous studies (Figures 1B,C). Additionally, the subjects with UC presented a higher and significant difference in IgA-bound bacteria compared to the healthy controls. Meanwhile, the subjects with CD showed more statistically significant differences in IgG-bound bacteria.

**TABLE 1:** Basic characteristics of healthy controls and IBD patients.

Cohort	CD	UC	Non-IBD
Number of patients	18	43 (25+18)	34
Number of specimens	18	43	34
% Male	16.60%	56%	50%
Age	31 ± 14.32	43 ± 14.50	37 ± 13.41
**Treatment**			
%Steroids (oral/IV)	11.10%	3.00%	0
%TNFα inhibitors	33.30%	0	0
%5-ASAs	72.20%	94%	0
%Other immunomod-modulators	5.50%	14%	0
% Antibiotics	16.60%	20%	0
%FMT	0	40% (18/43)	0

Similar to previous studies, we also evaluated free-fecal IgA and IgG by ELISA. The free-fecal IgA and IgG were higher in the patients with IBD than in the healthy controls (Figures 1D,E). These results suggested that the higher percentage of Ig-bound bacteria might be caused by the upregulation of free-fecal Ig. Previous studies have shown enhanced IgA release in the intestine under inflammatory conditions, which might be caused by increased IgA production by B cells or overexpression of the PIGR receptor and the polymeric immunoglobulin receptor in intestinal epithelial cells (MacDermott et al., 1986). The polymeric immunoglobulin receptor (pIgR) has dual functions in serving as the precursor of secretory component, which can enhance the immune functions of SIgA and transportation of locally produced dimeric IgA across mucosal epithelia (Johansen and Kaetzel, 2011). The higher detection of free-fecal IgG in the intestinal lumen might be due to the barrier breach in the intestine of patients with IBD.

### The Percentage of Immunoglobulin-Bound Bacteria Is Correlated With Inflammatory Bowel Disease

**FIGURE 2:**
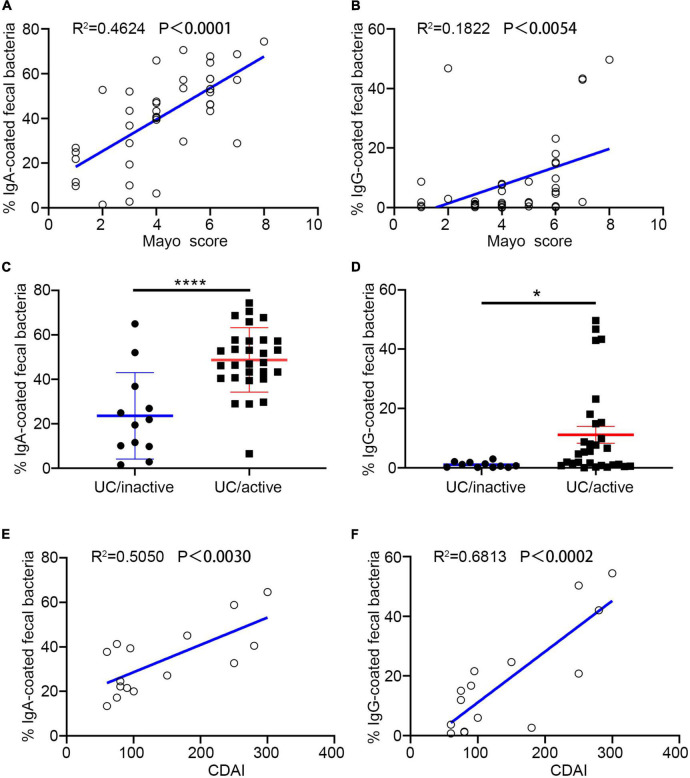
Percentage of Ig + fecal bacteria correlate with IBD activity. **(A,B,E)** Percentage of IgA + and IgG + fecal bacteria is correlated with ulcerative colitis (UC) activity. **(C,D,F)** Percentage of IgA + and IgG + fecal bacteria is correlated with CD activity. *p < 0.05, ****p < 0.0001, ns means no significance difference.

Both UC and CD are chronic remittent inflammatory diseases but present a distinct histopathology and different epidemic areas. Lesions in UC usually remain superficial and extend proximally, while CD has greater mortality (Cosnes et al., 2011; Olén et al., 2020). In this study, we separately estimated the correlation between the percentage of Ig-bound bacteria and disease activity. In the subjects with UC, we observed a significant correlation between the percentage of IgA/IgG + bacteria and disease activity scores (measurement score: Mayo) (Figures 2A,B). Compared to the percentage of IgA +, IgG + showed a stronger association with UC activity (Figures 2C,D). Furthermore, we found that the free-fecal IgA had a positive correlation with the percentage of IgA +, and that the free-fecal IgG was positively correlated to IgG + (Supplementary Figures 1A,B). The differences in our current results, compared to previous studies, might be associated with the expanded samples, different geographic locations, and dietary habits. We hypothesized that there is a positive correlation between free fecal Ig and the percentage of Ig-bound bacteria. As the main component of humoral immunity, IgG is found in extracellular fluids and blood, and its entry into the intestinal tract might occur through a barrier breach. Increased IgA might be related to increased production of IgA. Altogether, these results provided evidence that IgA/G-bound bacteria might be a good assessment measure in predicting the disease activity in UC, and that IgG + might be more beneficial than IgA +.

In the cohort, we enrolled 18 patients with CD and analyzed their fecal samples by flow cytometry. We found a significant positive correlation between the percentage of IgA + and IgG + bacteria with CD activity (measurement score: CDAI). Similar to UC, IgG + showed a stronger positive correlation with disease activity (Figures 2E,F). However, Ig-bound bacteria did not present statistical significance with fecal-free Ig (Supplementary Figures 1C,D). The distinct region of the lesion and pathological changes might be one of the reasons for these results. These data suggested that both IgA + and IgG + bacteria might be a non-invasive and promising measure in assessing disease activity in previously diagnosed patients with IBD, especially IgG +.

### 16S rDNA Sequencing Results of Immunoglobulin-Bound and Unbound Bacteria

Similar to previous studies, we investigated the taxa of bacteria in our IBD samples using 16S rDNA after sorting by flow cytometry. We provided the complement of the Asian IBD statistics in which the researched cohorts are primarily composed of patients with UC, which might be informative for future studies. The Ig-bound and unbound bacteria in each group presented different intersections in the Flower and Venn figures (Supplementary Figures 2A,B), possibly because anti-bacterial Ig antibodies are polyreactive (Bunker et al., 2017). The low affinity and widespread binding of commensal bacteria might overlap the high affinity and specific binding of pathogenic bacteria. We also found that the IgA/G bound bacteria in UC or CD have intersections, and that the IgA + and IgG + bacteria have partial intersections. Although all groups have a varied intersection, we detected specific IgA/IgG-targeting bacteria.

**FIGURE 3:**
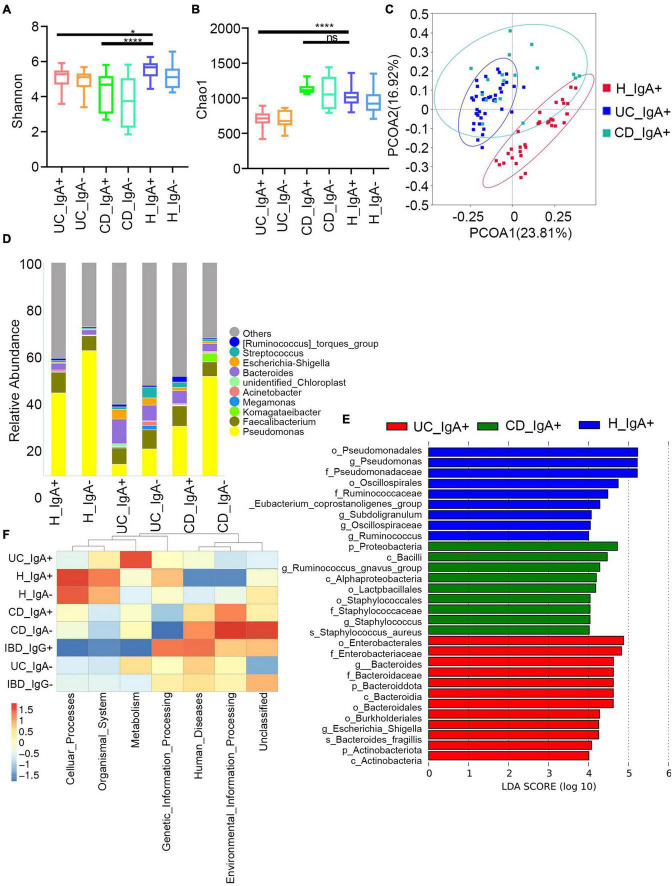
Characteristics of IgA -bound commensal bacteria. **(A)** Shannon index. **(B)** Chao1 index. **(C)** PcoA index. **(D)** Heatmap analysis of the difference in genera of the gut microbiota in each group of IgA^+/−^ by Metastat. **(E)** Stacked bar plot of the genus structure in each group. **(F)** Heatmap analysis of the function prediction among different groups.

Then, we assessed the broad diversity of Ig-targeted and untargeted bacteria between the IBD and healthy subjects. The Chao1 and Shannon indexes indicate the richness and diversity of OTUs (Figures 3A,B). Similar to previous studies, we observed high coincidence in IgA + and IgA- in both patients with IBD and healthy controls, and there were no significant differences between the Ig + and Ig– fractions from a given disease group. Moreover, we found higher α-diversity in both IgA + and IgA- from the healthy subjects compared to the patients with IBD. The healthy group was also separated by principal coordinate analysis (PCoA) (Figure 3C). These data are consistent with previous characterizations of the total microbiota in healthy subjects and subjects with IBD. One of the significant elements in IBD development is microbiota disorder and elevated adaptive immune response against intestinal bacteria.

Next, we analyzed the taxa abundance of IgA/G-bound bacteria. The top 10 genera and species in the cohorts are shown in Figure 3D. Compared to the healthy control (HC) group, increased IgA + bacteria were detected at the genera level in both UC and CD and comprehended *unidentified_Chloroplast*, *Escherichia-Shigella*, *Bacteroides*, *Streptococcus*, and *(Ruminococcus)_torques*_group. The taxon that only increased in UC was *Megamonas*, and in CD it was *Komagataeibacter*. Meanwhile, they presented a decreased abundance of *Pseudomonas*, *Faecalibacterium*, and *Acinetobacter.* We also observed an increased abundance of IgA + bacteria at the species levels in both UC and CD subjects, including *Faecalibacterium prausnitzii, Lolium perenne, Bacteroides fragilis, Anaerococcus vaginalis*, and *Oryza meyeriana.* The species that only increased in HC were *Ralstonia pickettii, Roseburia intestinalis*, and *Dialister* sp. *Marseille-P5638*, and in UC it was *Anaerostipes hadrus*. On the other hand, there were no species that only increased in CD (Supplementary Figures 2C,D). Since most IgG + bacteria in the flow cytometric sorting did not reach the 16S quality control, IgG + bacteria were uniformly analyzed without separating UC and CD. Regarding the IgG-bound bacteria, all genera overlapped with the IgA + taxa. Meanwhile, at the species levels, the increased abundance overlapped only for *Faecalibacterium prausnitzii* and *Anaerococcus vaginalis* (Supplementary Figure 3). These results showed that the Ig-bound was not specific, and that most bacteria were simultaneously targeted by both Ig. Next, we found that the taxa abundance of Ig- also overlapped with most Ig + bacteria. However, the degree of elevation in taxa abundance of Ig + bacteria was higher than Ig-(Figure 3D). Therefore, we focused on the Ig + bound bacteria rather than the Ig- ones.

According to the frequency and abundance of species between groups, we analyzed the indicator species by MetaStat and LEfSe. We conducted MetaStat to perform hypothesis testing on the species abundance data between groups to obtain the *p* and *q*-values. Finally, species with significant differences were screened according to *q*-value (Supplementary Figure 4A). At the genus level, *Fenollaria*, *Unidentified_Chloroplast*, and *Bifidobacterium* were significantly increased in the IgA + UC cohorts and did not entirely overlap with the IgA- UC cohorts. These findings revealed that IgA targeting reflects the immune recognition of bacteria independently of their taxonomic abundance. Then, we conducted LEfSe to find biomarkers with statistical differences between groups (Figure 3E). *Staphylococcus aureus* and *Bacteroides fragilis* were the biomarkers for CD and UC, respectively. We also observed that the biomarkers were mainly concentrated in the *Bacteroidota* and *Actinobacteriota* phyla in UC and *Proteobacteria* in CD.

Finally, we predicted the function of the microbiome (Figure 3F). The IgA + and IgG + bacteria in IBD mainly predicted the disease occurrence function, and the correlation of IgG + was stronger. This might be because an increased proportion of IgG + bacteria was not observed in the healthy subjects and other diseases such as liver and rheumatic immune diseases. The high levels of IgA + in the healthy controls were mainly caused by some symbiotic bacteria related to the cell cycle (Supplementary Figure 4B). Previous studies have also shown that the IgA + in healthy people can primarily combine with symbiotic bacteria (Bunker et al., 2017).

### Immunoglobulin A-Targeted Bacteria Pre and Post Fecal Microbiota Transplantation

**FIGURE 4:**
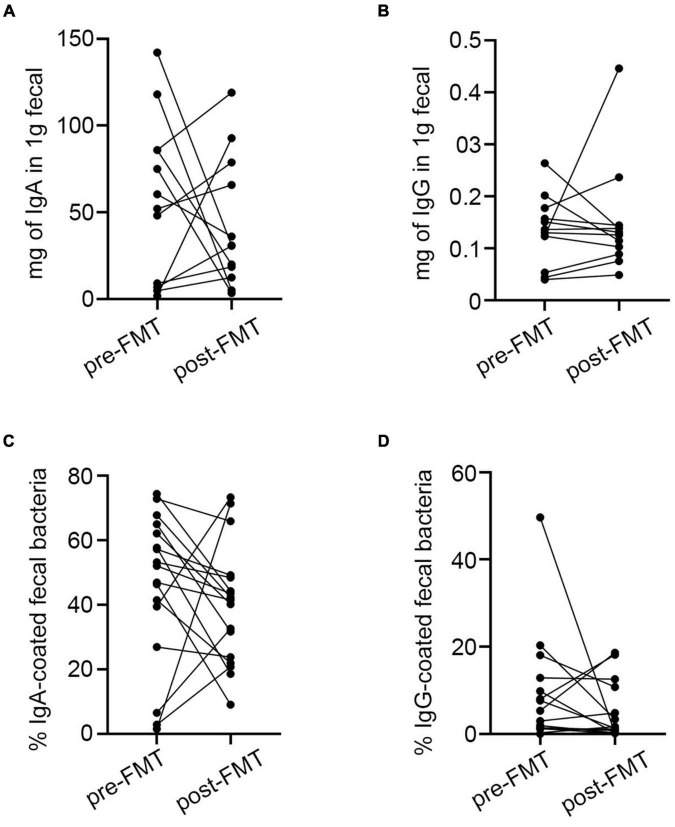
Differences in IgA/G-bound microbiota and fecal free Ig before and after FMT. **(A,B)** Fecal-free Ig before and after FMT. **(C,D)** IgA/G-bound microbiota before and after FMT. *p < 0.05, ****p < 0.0001, ns means no significance difference.

**FIGURE 5:**
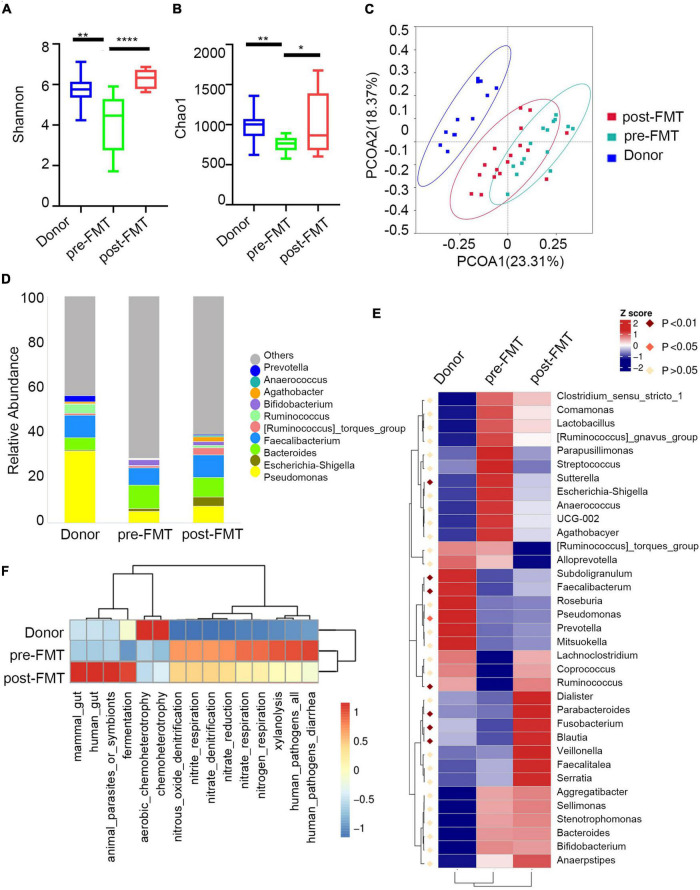
Characteristics of IgA -bound bacteria before and after FMT. **(A)** Shannon index. **(B)** Chao1 index. **(C)** PcoA index. **(D)** Heatmap analysis of the difference in genera of the gut microbiota pre/post FMT by Metastat. **(E)** Stacked bar plot of the genus structure in pre/post FMT. **(F)** Heatmap analysis of the function prediction among pre/post FMT. *p < 0.05, **p < 0.01, ****p < 0.0001, ns means no significance difference.

In the 18 patients who accepted FMT therapy, the fecal-free IgA/G did not differ between pre- and post-transplant samples (Supplementary Figures 5A,B). The percentage of IgA/G-bound bacteria also did not differ before and after FMT treatment (Supplementary Figures 5C,D). However, most of the post-FMT samples presented a decrease in Ig-bound bacteria as well as free fecal Ig contains compared to the pre-FMT group (Figures 4A–D). The Anosim analysis is a non-parametric test that can evaluate whether the difference between groups was significantly greater than the difference within the group to determine whether the grouping was meaningful. Thus, we tested the significance of the difference between groups based on the rank of the Bray-Curtis distance value and found that the pre and post-FMT groups were comparable (Supplementary Figure 6A). Moreover, we found that after FMT, the diversity of IgA-binding bacteria was higher compared to pre-FMT, which was reflected by increases in Chao1 and Shannon indexes (Figures 5A,B). Furthermore, according to the PCoA, extensive changes in IgA targeting were detected before and after FMT (Figure 5C). Before FMT, the IgA-targeted bacteria resembled the results described above. However, we found that at the genus level, the abundance of *UCG-002, Agathobacter, Ruminococcus_torques_group*, and *Subdoligranulum* targeted by IgA was increased after FMT, while *Serratia* was decreased (Figures 5D,E). Additionally, we observed a poor correlation between IgA donors targeting for the UC microbiota before FMT. Although FMT can make the patient’s microbiome present a donor tendency, some bacteria such as *Escherichia-Shigella* were highly bound to IgA before FMT. The number of donors targeted by the bacteria was extremely low and was not completely recovered to the donor status after FMT (Figure 5D). Generally, rising bacteria are a common IgA target in healthy adults, and decreases in the aforementioned bacteria are also common in patients with UC with increased IgA abundance (Figure 5F). Hence, FMT could lower the Ig-bound ability by rebuilding the intestinal microbiota in patients with IBD.

### Response of Immunoglobulin A to Different Delivery Methods

The patients enrolled in this study received FMT by gastroscopy spraying or colonic TET. The responses of IgA in the colon and small intestine are very different. Therefore, the route of delivery might change the initiation of the immune response. Thus, we tested the significance of different delivery methods based on Anosim analysis and found no significant difference between gastroscopy spraying and colonic TET, which was also demonstrated in the PCoA (Supplementary Figures 6B,D). Then, we verified whether there were significant differences in the interaction between IgA and bacteria based on the method of delivery. Both gastroscopy and colonic TET recipients had comparable IgA-coating levels of the microbiota (Supplementary Figure 5E). By analyzing the average relative abundance and bacteria biomarkers in post-transplant patients based on delivery method, we found that no harmful taxa were significantly different between gastroscopy and colonic TET recipients (Supplementary Figures 7A,B). These data indicated that the delivery route might not affect the fecal IgA-bacteria interactions after FMT.

### Response of Immunoglobulin A to Different Donors

**FIGURE 6:**
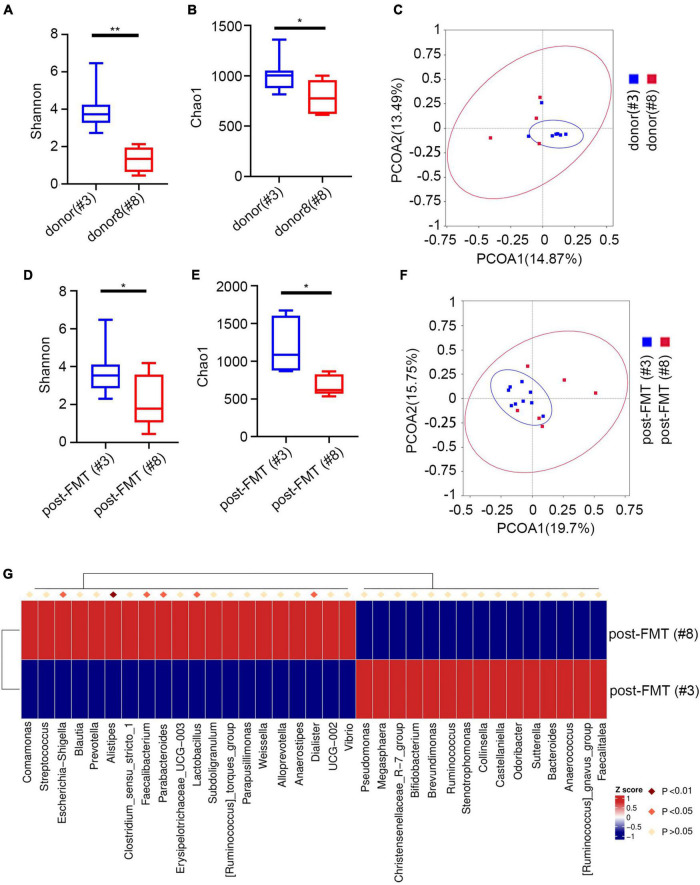
IgA-targeting of microbiota post-FMT is influenced by donor microbiota. **(A)** Shannon index of different donors. **(B)** Chao1 index of different donors. **(C)** PcoA index of different donors. **(D)** Shannon index of patients post FMT by different donor. **(E)** Chao1 index of patients post FMT by different donors. **(F)** PcoA index of patients post FMT by different donor. **(G)** Heatmap analysis of patients post FMT by different donors by Metastat. *p < 0.05, **p < 0.01.

Next, we evaluated the relationship between pre/post-FMT and donor samples. Since the IgA-targeted bacteria of the recipient and the donor were similar, we explored whether the IgA targeting of the recipient was related to different donors. The cohort used the feces of two different donors. Nine patients with UC received the feces of donor #3, and 6 patients with UC received the feces of donor#8. We did not find significant differences in the proportion of IgA bound to bacteria in FMT recipients regardless of the donor (Supplementary Figure 6C). Nevertheless, the total microbiota between the recipients of two different fecal donors was significantly different (Supplementary Figure 5C). The PCoA of IgA-targeted bacteria showed that the donor’s microbiota overlaps with the FMT recipient, and that most of them presented similar IgA-targeted states (Figures 6C,F). After FMT, the patient restored the relative taxa abundance and high IgA recognition. Furthermore, we found significant changes in Chao1 and Shannon diversity indexes between different FMT donors (Figures 6A,B) and the acceptors from different FMT donors (Figures 6C–E). The PCoA of IgA-targeted bacteria showed that the donor’s microbiota overlaps with the FMT recipient, and most of them presented similar IgA-targeted states (Figures 6C,F). Additionally, we detected significant changes in the relative abundance of many strains between post-FMT from different recipients (Figure 6G). For example, more *Escherichia-Shigella* was detected in the post-FMT recipient from donor 8. Although different sex groups have different immune responses, the donors used in this study were all male. Therefore, the driving factors leading to changes in IgA targeting after FMT might not be related to sex. These data indicated that after FMT, the IgA targeting of the human microbiota was significantly affected by the IgA targeting of the donor microbiota.

### Establishment of Dextran Sulfate Sodium Colitis Mice Model and Fecal Microbiota Transplantation Intervention

**FIGURE 7:**
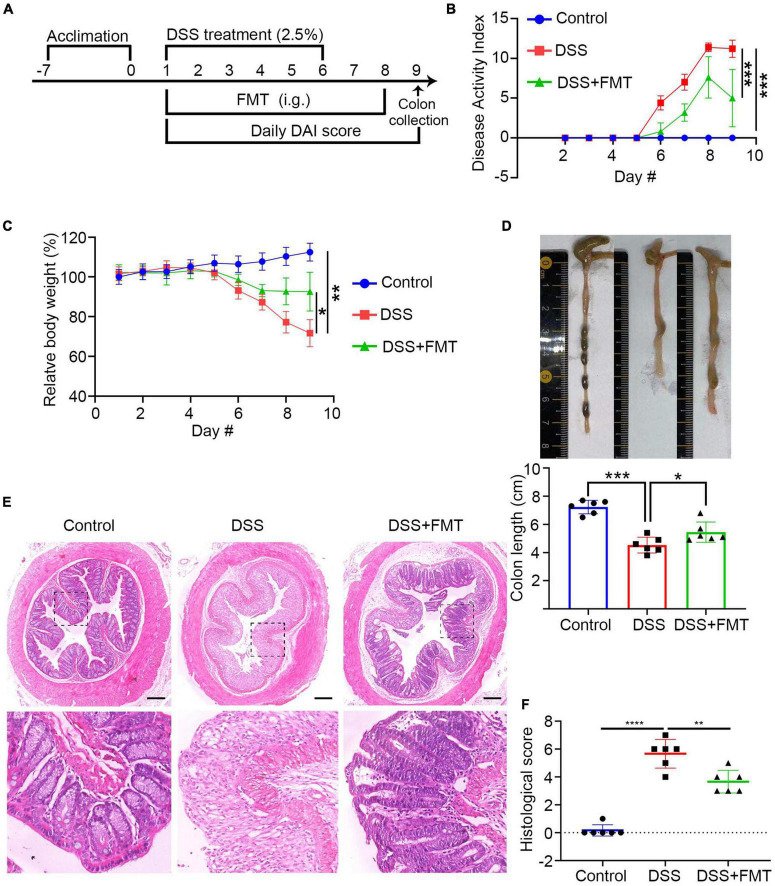
FMT with the gut microbiota from donor 3 or mice significantly ameliorates clinical symptoms and inflammation in colitis mice. BALB/c mice were randomized and fed with normal water as the control or water containing 2.5% DSS to induce colitis. The control and some DSS-fed mice (DSS group, *n* = 6) were fed with PBS, whereas other DSS-fed mice were fed with fresh human or microbiota as the DSS + FMT group (*n* = 6 per group). **(A)** Schematic diagram of the experimental design. **(B)** The dynamics of Disease Activity Index (DAI) in each group. **(C)** The relative body weight in each group. **(D)** Mouse colon lengths in each group. **(E)** H&E. **(F)** Histology of colonic tissue score in each group. Data are individual means or the mean ± SD of each group from three separate experiments. *p < 0.05, **p < 0.01, ***p < 0.001, ****p < 0.0001.

To understand the mechanisms underlying the FMT treatment and the relationship between FMT and IgA/G-targeted bacteria, we established acute DSS-induced colitis in mice and administered human fecal bacteria (donor source, consistent with previous experimental collections). The schematic diagram of the experimental design is shown in Figure 7A. After FMT, the colitis of acute DSS mice was relieved. This mitigation was mainly reflected by the following aspects: compared to controls, the DAI value of colitis mice increasingly fluctuated. Moreover, the DAI of the DSS + FMT group value was significantly lower than the DSS group at the end of the treatment (Figure 7B). The bodyweight of the DSS + FMT group was significantly higher than the DSS group (Figure 7C). Similarly, the colon was significantly longer in the DSS + FMT group than in the DSS group (Figure 7D).

The colon tissue analysis showed that the colon mucosa structure of control mice was clear, the epithelium was intact, the glands were orderly, the goblet cells were abundant, and there was no apparent inflammatory infiltration in the lamina propria (Figure 7E, left). The DSS mice showed severely damaged and few epithelial cells, incomplete glands, and extensive spread of inflammatory infiltration, which is a sign of inflammatory colon injury (Figure 7E, middle). In contrast, these pathological changes were significantly reduced in the DSS + FMT group (Figure 7E, right). The quantitative analysis showed that, compared to the control mice, the histopathological score of the colon in the DSS group was significantly higher but reduced in the FMT-treated group (Figure 7F). ZO-1 is a crucial tight junction protein that controls the integrity of the colonic epithelium. Since inflammation destroys the integrity of the colonic epithelium and the function of the intestinal epithelial barrier (Yin et al., 2015), we evaluated the levels of ZO-1 in the colon tissues of different mice groups by immunofluorescence. The levels of ZO-1 in the colon tissue of the DSS group were significantly lower than those of control mice but partially recovered in the FMT-treated mice (Supplementary Figure 8A). Additionally, inflammation can also lead to destruction of goblet cells, reducing mucin secretion. The immunofluorescence of muc2 indicated that its expression in intestinal tissue from the DSS group was significantly lower compared to the control and FMT groups (Supplementary Figure 8B).

### Fecal Microbiota Transplantation Can Decrease Immunoglobulin A/Immunoglobulin G + B Cells in the Intestine of Dextran Sulfate Sodium Mice

**FIGURE 8:**
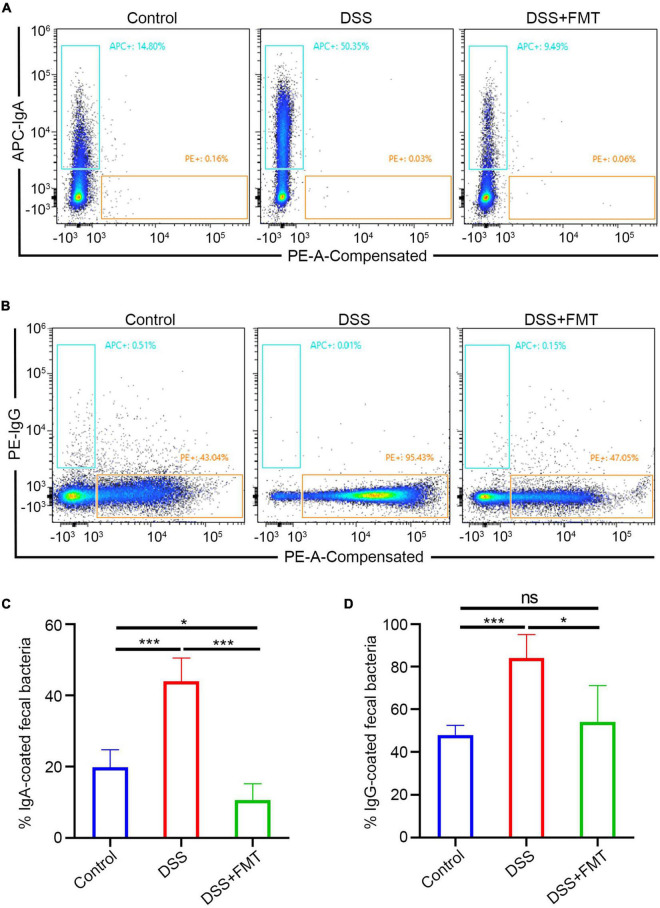
IgA/G + gut bacteria were determined by flow cytometry. **(A,C)** Fecal IgA + bacteria by FACS. **(B,D)** Fecal IgG + bacteria by FACS. *p < 0.05, ***p < 0.001, ns means no significance difference.

The above experiments mainly showed that donor human fecal bacteria can relieve acute colitis in mice, consistent with clinical practice. Regarding the IgA/G-seq in mouse feces, we observed similar results to human samples, that is, the proportion of IgA/G-targeted bacteria in DSS-induced colitis mice was higher than in controls. However, unlike human samples, the proportion of IgA/G-targeted bacteria was reduced to approximately normal levels in the mice model. IgA-targeted bacteria are shown in Figure 8A, IgG-targeted bacteria are shown in Figure 8B, and the quantitative analysis is presented in Figures 8C,D. Whether this increase in the proportion of Ig-targeted binding bacteria was due to increased production of IgA/G + B cells or the upregulation of receptors for IgA transport or destruction of the barrier leading to the leakage of immunoglobulins remained unclear.

**FIGURE 9:**
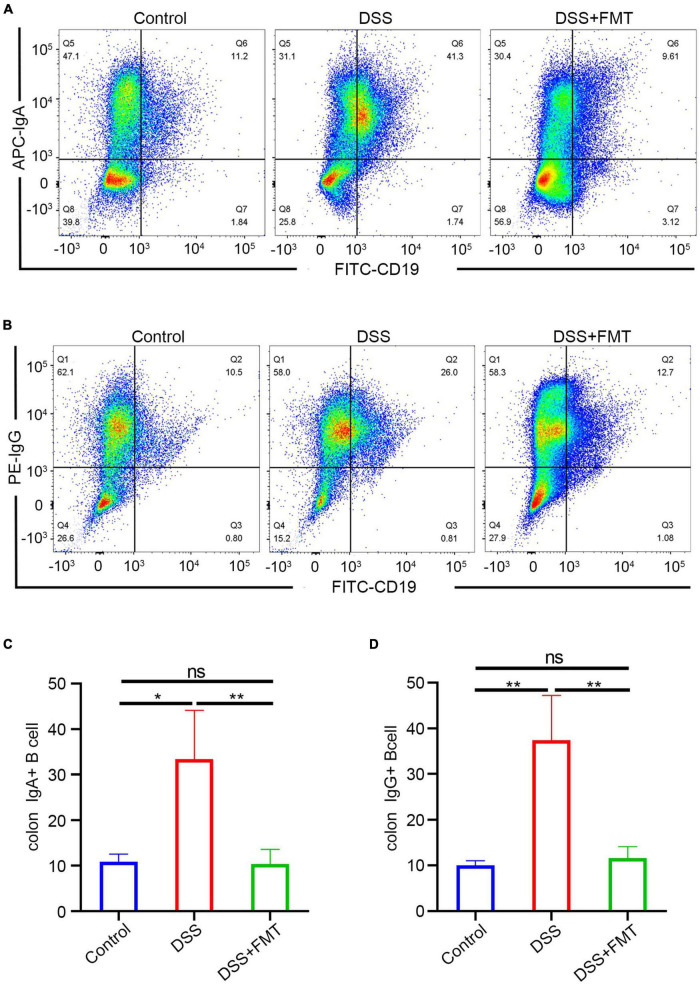
IgA/G memory B cells of the intestine were determined by flow cytometry. **(A,C)** IgA memory B cells by FACS. **(B,D)** IgG memory B cells by FACS.

Hence, we quantitatively detected the IgA/IgG + B cells in mouse intestinal tissue by flow cytometry. The FACS of IgA + B cells is presented in Figure 9A and that of IgG + B cells in Figure 9B. These results showed that the number of IgA/IgG + B cells in the DSS enteritis mice was significantly increased compared to the controls (Figures 9C,D). Moreover, compared to the DSS group, FMT treatment led to a significantly lower proportion of IgA/IgG + B cells. Furthermore, no significant differences in IgA/G + B cells were detected in the spleen (Supplementary Figures 9A–D). In summary, we consider that the increase in the proportion of Ig-targeted binding bacteria might be derived from the increased expression of IgA/IgG + B cells. However, the upregulation of receptors for IgA transport or immunoglobulin leakage into the intestine due to destruction of the intestinal barrier cannot be ruled out.

## Discussion

In this study, we analyzed IBD and UC fecal samples before and after FMT by flow cytometry and 16S rDNA sequencing. First, we showed that the percentage of IgA/G bound bacteria in UC and CD was increased compared to the healthy subjects. This result indicated that the commensal bacteria of patients with IBD had strengthened their adaptive immune responses, consistent with previous studies. Furthermore, the increased percentage of IgA and IgG binding was related to disease activity. Compared to the healthy subjects, the percentage of IgA and IgG binding was increased by 1–3 times and several tens of times, respectively. Regarding the 16S rDNA sequencing, we found that specific taxa such as *Megamonas* and *Acinetobacter*, presented increased Ig targeting. For patients with UC undergoing FMT therapy, the treatment could help rebuild the intestinal pattern of IgA-targeted bacteria and tended to the donor profile. No significant differences were detected in the abundance and taxa of IgA-bound bacteria in post-FMT recipients based on different delivery methods. Finally, by establishing a DSS colitis model and after FMT, we observed that FMT might downregulate the production of IgA/G + B cells and reduce the percentage of IgA/G-bound bacteria.

Our current results were similar to previous studies. The percentage of IgA and IgG-bound bacteria was increased in the patients with IBD and was positively correlated with disease activity. Consistent with a previous report, we also found that increased IgG-bound bacteria were only detected in subjects with IBD. Additionally, we observed that the free-fecal IgA and IgG in IBD were increased, and that there was a correlation with the percentage of IgA/G binding. The free-fecal IgG was more positively correlated with IBD than IgA. However, there are contradictions between our fecal-free IgA results and previous studies. These differences might be related to differences in sample size and geographic location. Increased fecal-free Ig was mainly related to the adaptive immune response, leading to increased secretion of memory B cells or barrier destruction of the intestinal lumen, finally resulting in IgG leakage. Additionally, we observed an increase in fecal-free IgA in cirrhosis and rheumatic diseases different from IgG.

Considering that the IgG increases in this study were mainly due to the destruction of the mucosal barrier, we found that the IgG-targeting bacteria were not detected in other diseases such as liver cirrhosis or cirrhosis with its complications, and rheumatism including rheumatoid arthritis and systemic lupus erythematosus (These results have not been proved). Compared to the healthy subjects, the percentages of IgA and IgG binding were 1–3 times and dozens of times higher, respectively. Altogether, these data suggested that IgG-bound bacteria might be more specific for IBD research.

Regarding the sequencing of IgA/G-binding bacteria, we supplemented the results of the UC-based cohort and Asian patients. In the top 10 most abundant genera, besides three symbiotic bacteria (*Pseudomonas*, *Faecalibacterium*, and *Komagataeibacter*), we found that *Megamonas*, *Acinetobacter*, *Escherichia-Shigella*, *Streptococcus*, *Fenollaria*, *Bacteroides*, and other bacteria presented an increased percentage of Ig binding. Previously, an increased abundance of *Escherichia-Shigella* and *Bacteroides* was detected in patients with IBD, consistent with our current results. However, no increase in *Megamonas*, *Acinetobacter*, and *Streptococcus* was observed. Therefore, our results indicated that increased Ig binding to streptococci is not related to the appearance of dysbiosis but might reflect the induction of antigen-specific responses during IBD. Increased Ig binding to *Streptococcus* in IBD has also been reported in previous studies. Here, we found that increased Ig binding to *Acinetobacter* (except *Streptococcus*) indicates that the immune interaction with oral bacteria might play an essential role in IBD pathogenesis. Additionally, we found that *Megamonas* was mainly increased in UC response, and that *Acinetobacter* was mainly increased in CD. *Megamonas* is not the dominant intestinal bacterium of Europeans and Americans, but it is found in Chinese, comprehending a characteristic bacteria of Asians. Moreover, the human genome detected by metagenomic sequencing is significantly higher in individuals with colorectal cancer (CRC) in all stages compared to healthy individuals (Viladomiu et al., 2017). However, the pathogenic role of *Acinetobacter* and *Megamonas* in IBD has not been clearly defined. Furthermore, more IgA + *Staphylococcus aureus* was observed in the patients with CD and more IgA + *Bacteroides fragilis* and *Escherichia-Shigella* were detected in the patients with UC by LEfSe. *Escherichia coli* and *Bacteroides* are highly targeted by IgA in the inflamed intestine (Nguyen et al., 2010; Kau et al., 2015). However, there is no previous report on IgA-coated *Staphylococcus aureus* in patients with CD. It has been previously demonstrated that hospitalized patients with IBD are at increased risk of MRSA compared with non-IBD GI and general medical inpatients. Importantly, patients with IBD affected by MRSA have increased mortality (Han et al., 2020). Whether this phenomenon is associated with highly IgA-coated *Staphylococcus* remains unknown. Whole-genome sequencing of cultured isolates or Ig-combined partial metagenomic sequencing might help determine whether patients with IBD carry unique strains that can promote immune recognition and disease pathogenesis.

The sequencing analysis after FMT showed that the changes in IgA-bound bacteria resembled the donors, and that the delivery methods did not impact the IgA-targeted bacteria in the FMT recipients. Although the taxa in post-FMT patients presented similar IgA targeting status to pre-FMT, indicating that FMT cannot completely replace the recipient microbiome, we observed an increase in IgA binding to probiotics or symbiotic bacteria such as *Pseudomonas*, *Faecalibacterium*, *Ruminococcus*, *Bifidobacterium*, *Agathobacter*, and *Prevotella*. However, the classification differences between the donor and patient strains and the strain level still need to be resolved by deep metagenomic sequencing, and the bacterial identity can be used to better understand IgA-targeted mutations. Given the importance of IgA for intestinal homeostasis, a better understanding of these interactions and their recovery after FMT might help improve the rational design of microbial therapies for UC. Finally, since clinical FMT treatment is usually temporary rather than long-term, we also conducted experiments on mice to evaluate the underlying mechanisms. By constructing a DSS-induced enteritis model and after FMT, we found that the number of IgA/IgG + B cells in DSS colitis mice was significantly increased compared to the control group. Additionally, FMT might downregulate the production of IgA/G + B cells, decreasing fecal-free IgA/G, and, finally, reducing the percentage of IgA/G-bound bacteria.

However, there also exists several limitations. First, the sample size was limited, and the cohort was concentrated in one hospital. Second, the 16S sequencing results are indeterminate with regard to strains, and whole-genome sequencing of cultured isolates or Ig combined with partial metagenomic sequencing may help to identify patients with IBD with unique strains that may promote immune recognition and disease pathogenesis. Lastly, in order to observe the changes in fecal IgA-binding bacteria of patients with IBD after FMT, this study unified the factors that may affect the experimental results, such as transplantation dose, transplantation frequency, donor identity (2 donors), and transplantation route (2 types), which may increase the reliability of results. However, because of differences in donors/region/methodologies and the diversity of intestinal bacteria, it is difficult for the FMT system to achieve full standardization (Zhang et al., 2012). Therefore, standardization of FMT has become an urgent need of the time.

## Conclusion

In this study, Ig-seq was performed on the stool of patients with IBD and those with IBD who received FMT, and it was found that compared with healthy people, fecal IgA/G-binding bacteria in the patients with UC and CD are expected to be potential biomarkers for evaluating disease activity. Compared with the healthy group, the composition of intestinal flora in the patients with IBD was changed, and the abundance of IgA-bound pro-inflammatory bacteria was higher in the patients with IBD, while the abundance of IgA-bound commensal bacteria and probiotics was lower. In addition, we found that the IgA-targeting gut microbiota was reconstituted and tended to the donor state after FMT treatment in the patients with UC. Besides, the changes in IgA-binding microbiota were related to different donors, not to the delivery route. Finally, by establishing the DSS colitis mouse model and giving FMT treatment, it was found that the increase in the percentage of IgA-binding bacteria in DSS colitis mice may be related to the increase in IgA secretion, independent of pIgR expression. FMT can reduce the percentage of IgA-binding bacteria by downregulating IgA secretion.

## Data Availability Statement

The original contributions presented in this study are included in the article/Supplementary Material, further inquiries can be directed to the corresponding author/s.

## Ethics Statement

This study was approved by the Ethics Committee of Guangzhou First People’s Hospital (Permission K-2017-103-02). Informed consent was obtained from all patients for being included in the study. The patients/participants provided their written informed consent to participate in this study. The animal study was reviewed and approved by the Medical Ethics Committee of Guangzhou First People’s Hospital. Written informed consent was obtained from the individual(s) for the publication of any potentially identifiable images or data included in this article.

## Author Contributions

W-QH, H-LH, and Y-DL were involved in the design of the study, recruitment of the patients, and drafting of the article. W-QH was involved in statistical analysis, interpretation of the data, and drafting of the article. WP collected the patient clinical and follow-up information. Y-LZ and H-MX sorted the fecal samples into Ig ± by flow cytometry. WP and W-QH established the DSS model and gave FMT intervention. Y-QN and CZ designed, organized the study, and contributed to interpretation of the data, and revision of the article. All authors contributed to the article and approved the submitted version.

## Conflict of Interest

The authors declare that the research was conducted in the absence of any commercial or financial relationships that could be construed as a potential conflict of interest.

## Publisher’s Note

All claims expressed in this article are solely those of the authors and do not necessarily represent those of their affiliated organizations, or those of the publisher, the editors and the reviewers. Any product that may be evaluated in this article, or claim that may be made by its manufacturer, is not guaranteed or endorsed by the publisher.
