# Supplementary material for: Altered Pattern of Immunoglobulin A-Targeted Microbiota in Inflammatory Bowel Disease After Fecal Transplantation
Source: Front Microbiol. 2022 Jun 22;13:873018. doi: 10.3389/fmicb.2022.873018 (PMC9257281; doi:10.3389/fmicb.2022.873018)
Supplement: Supplementary file 1 [file Data_Sheet_1.pdf]

Supplementary Figure1

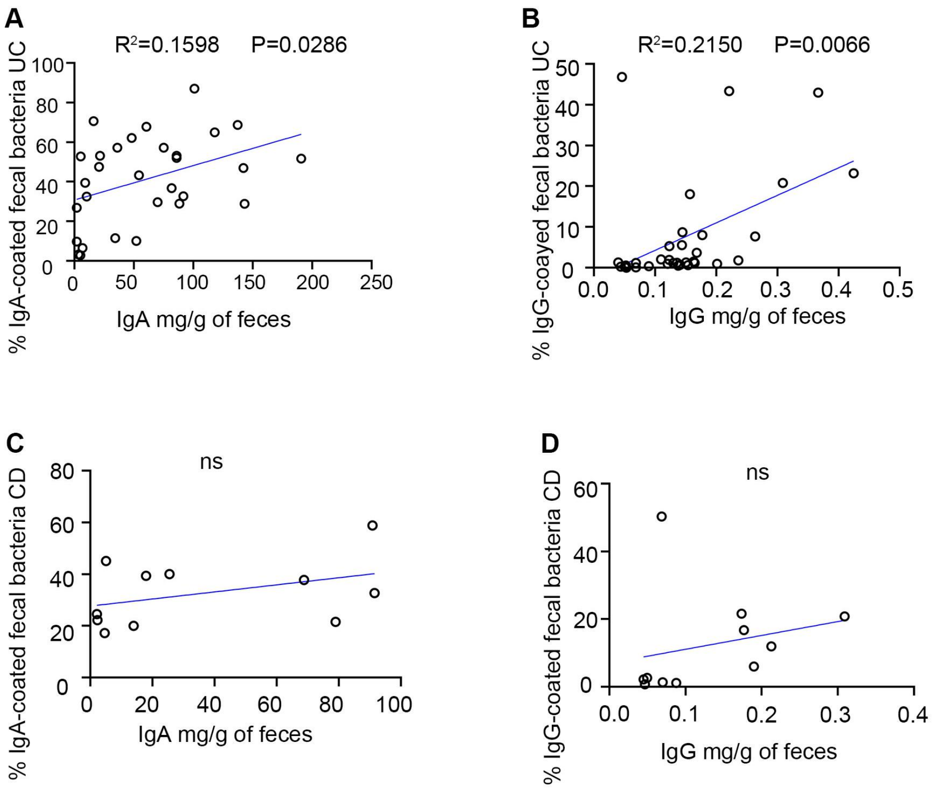

Supplement figure1

Relationship of free fecal Ig and percentage of Ig bound bacteria

Supplementary Figure2

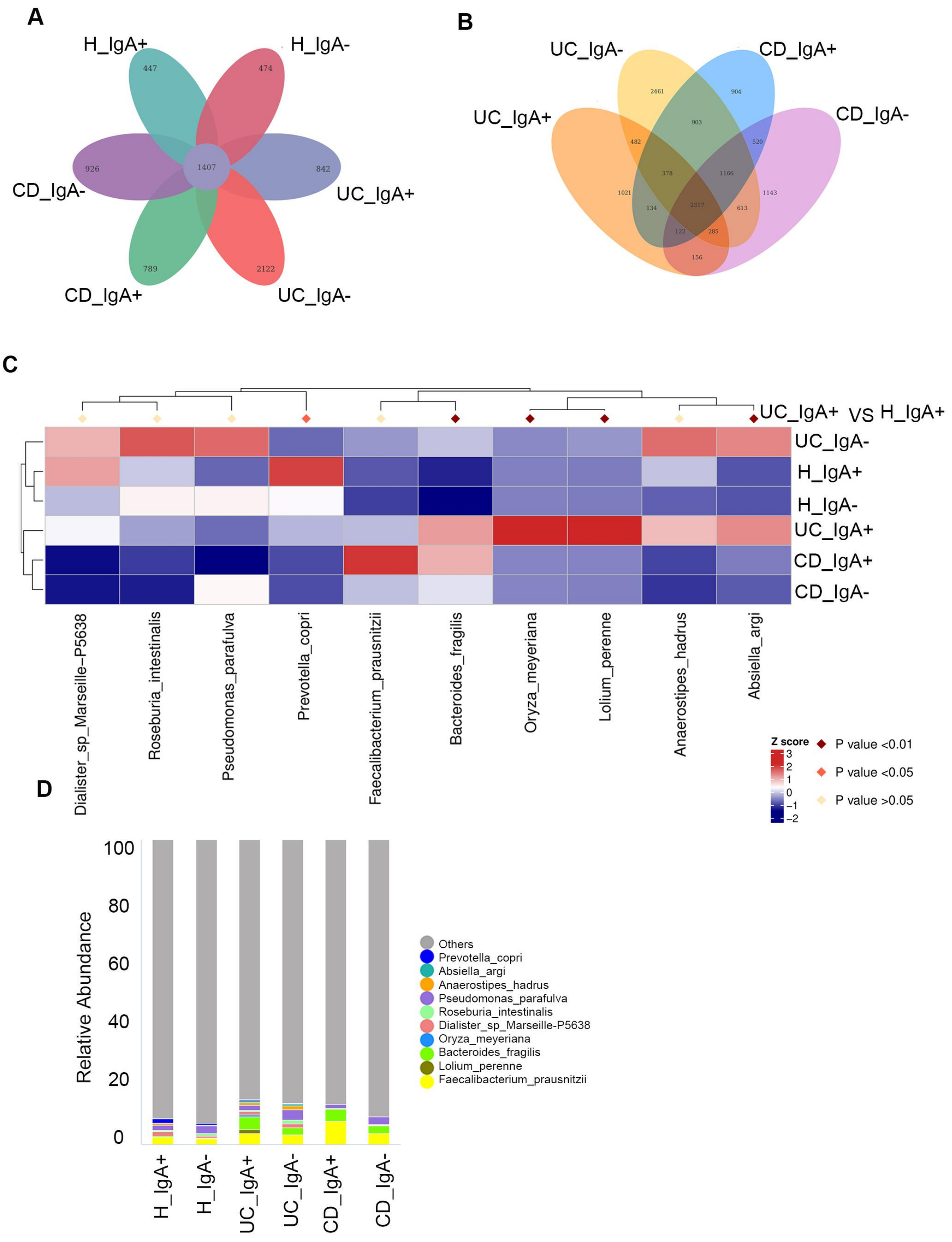

Supplement figure2

Characteristics of IgA -bound commensal bacteria A. Venn figure
B.Flowerfigure
C. Heatmap analysis of the difference in species of the gut microbiota by Metastat.
D. Stacked bar plot of the species structure in each groups.

A

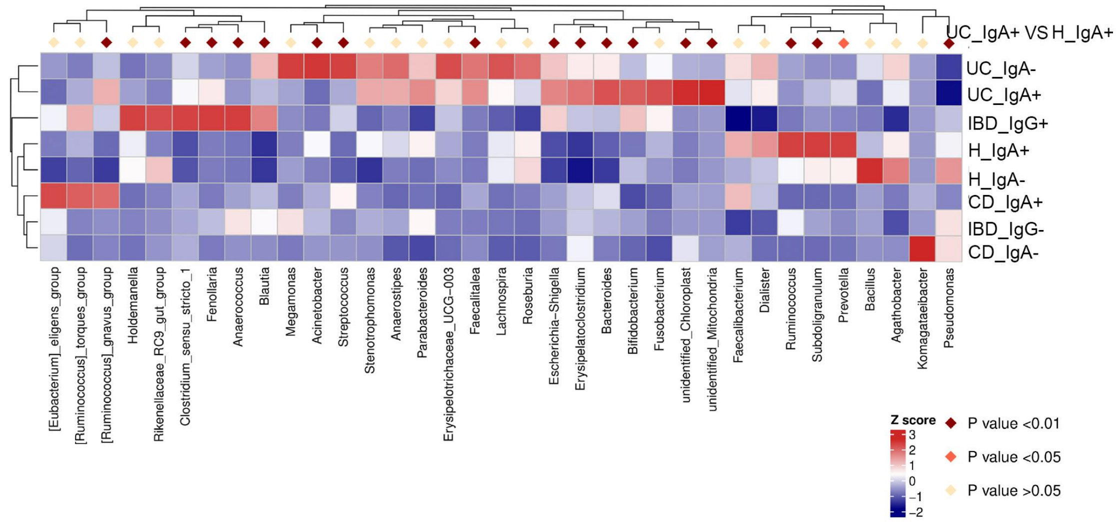

B

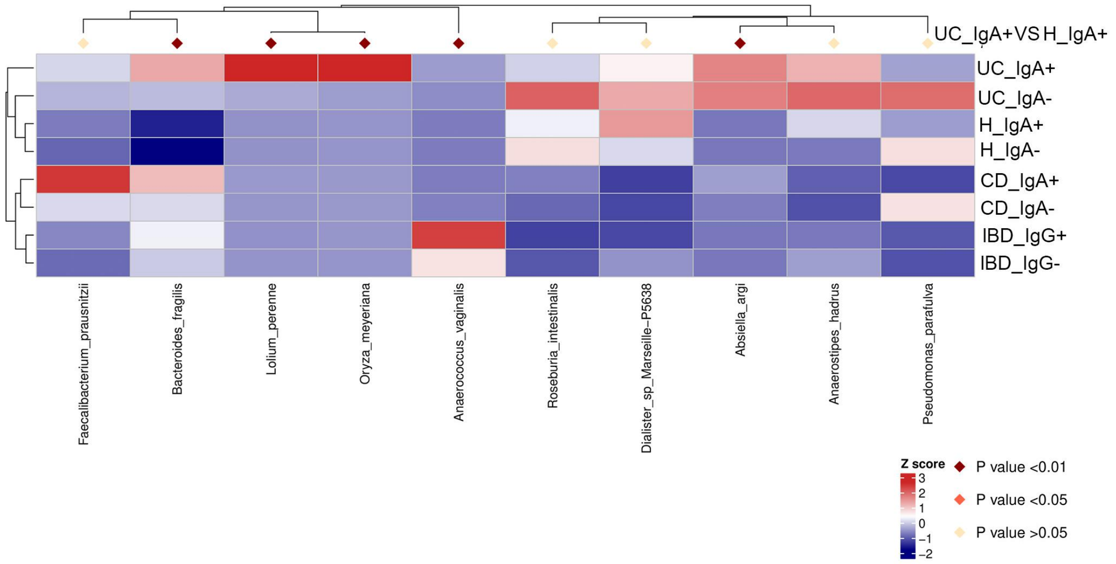

Supplement figure3

Characteristics of IgA and IgG -bound commensal bacteria

A. Heatmap analysis of the difference in genera.
B. Heatmap analysis of the difference in species.
C. Heatmap analysis of the function prediction.

SupplementaryFigure4

A

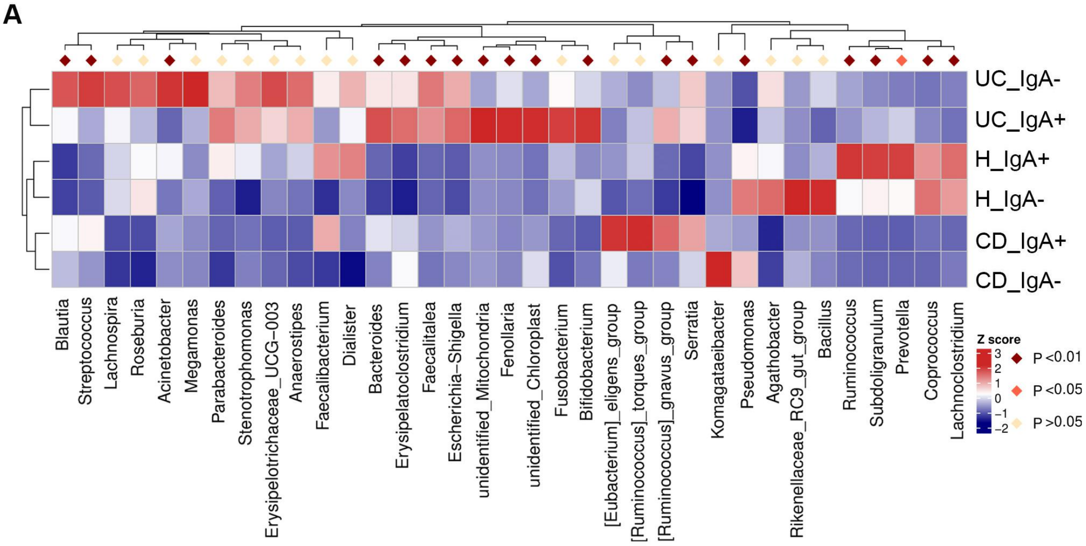

B

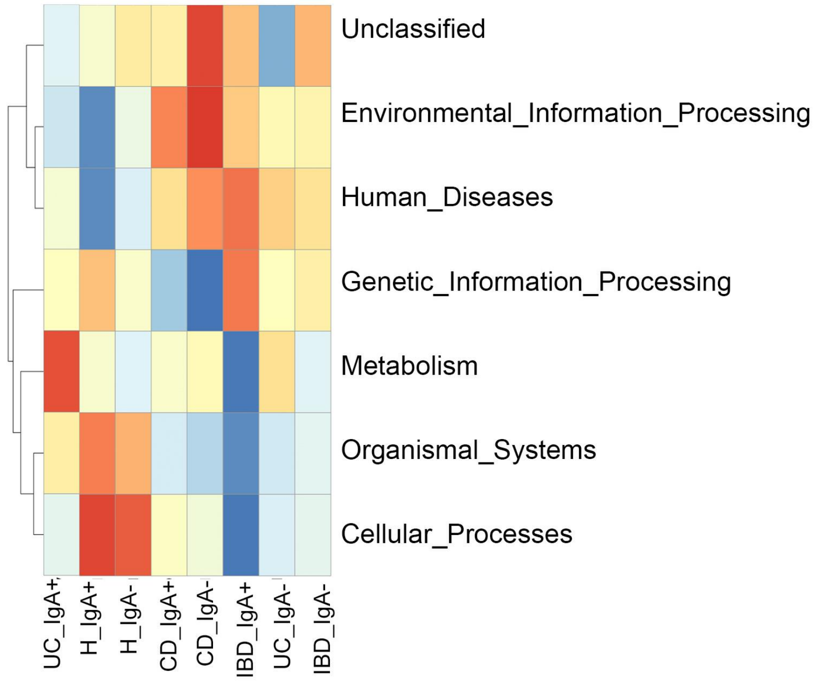

Supplement figure4

Characteristics of IgA -bound commensal bacteria

A.Heatmap analysis of the difference in species.

B.Heatmap analysis of the function prediction.

Supplementary Figure5

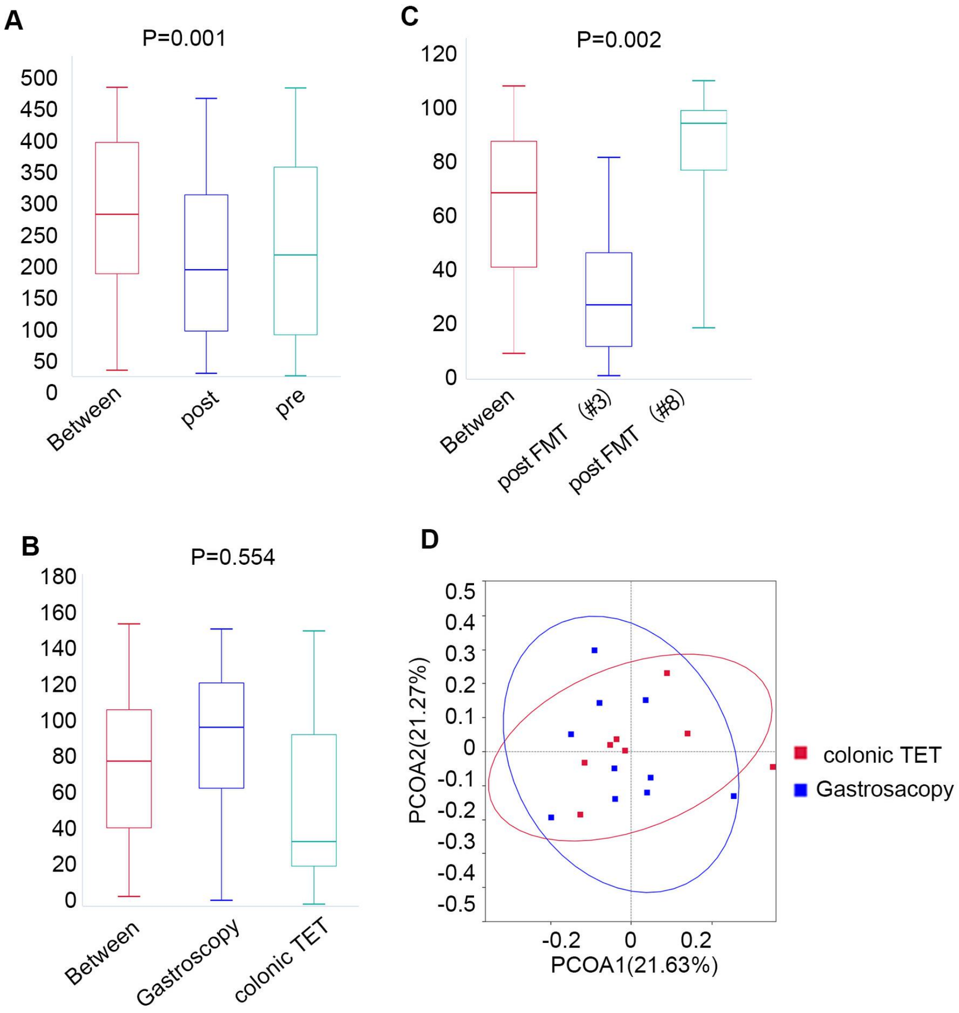

Supplement figure5

A. Anosim analysis of pre and post FMT
B. Anosim analysis of post FMT by different delivery method
C. Anosim analysis of post FMT by different donor.
D. PcoA of post FMT by different delivery method

Supplementary Figure6

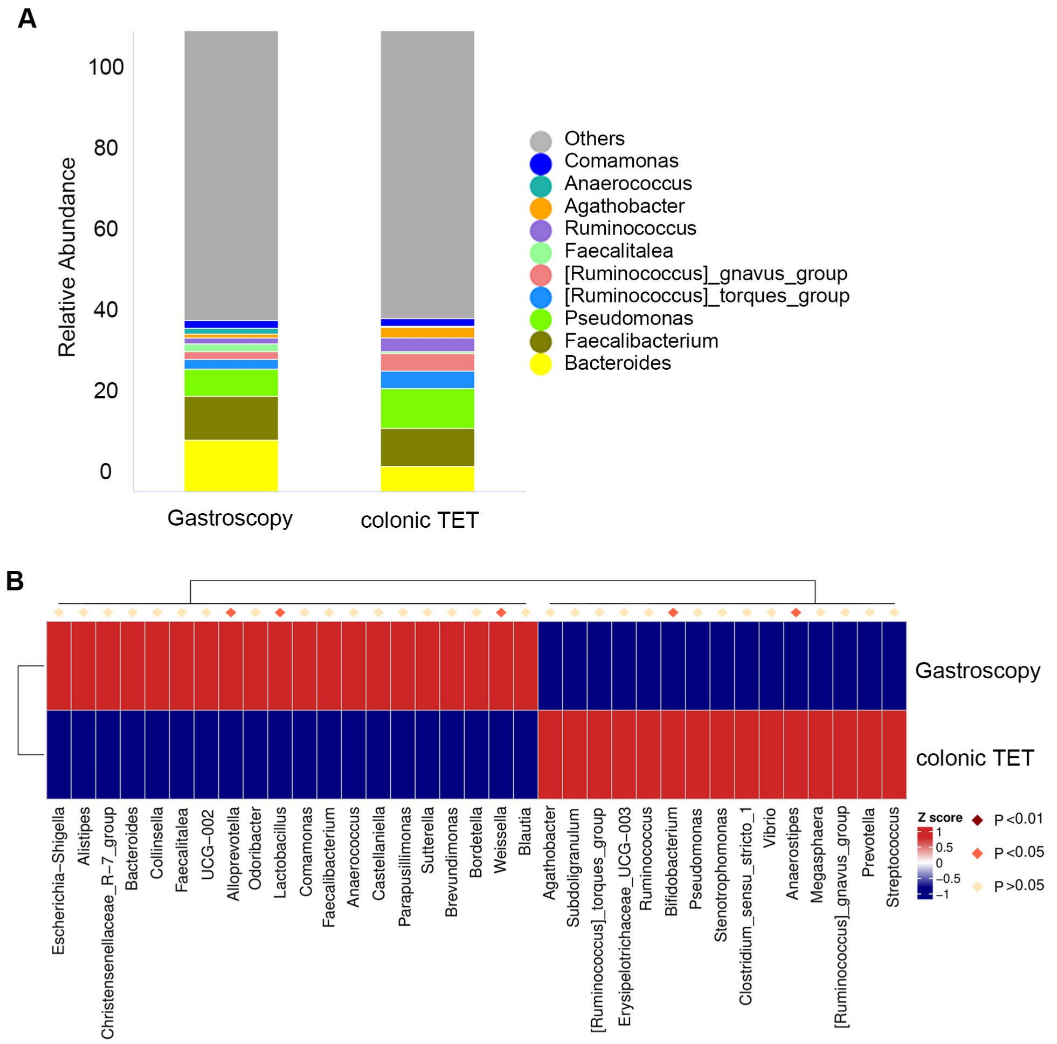

Supplement figure6

Characteristic of IgA bound bacteria of post FMT by different delivery method

A. Stacked bar plot of genera
B. Heatmap analysis of the difference in genera

Supplementary Figure7

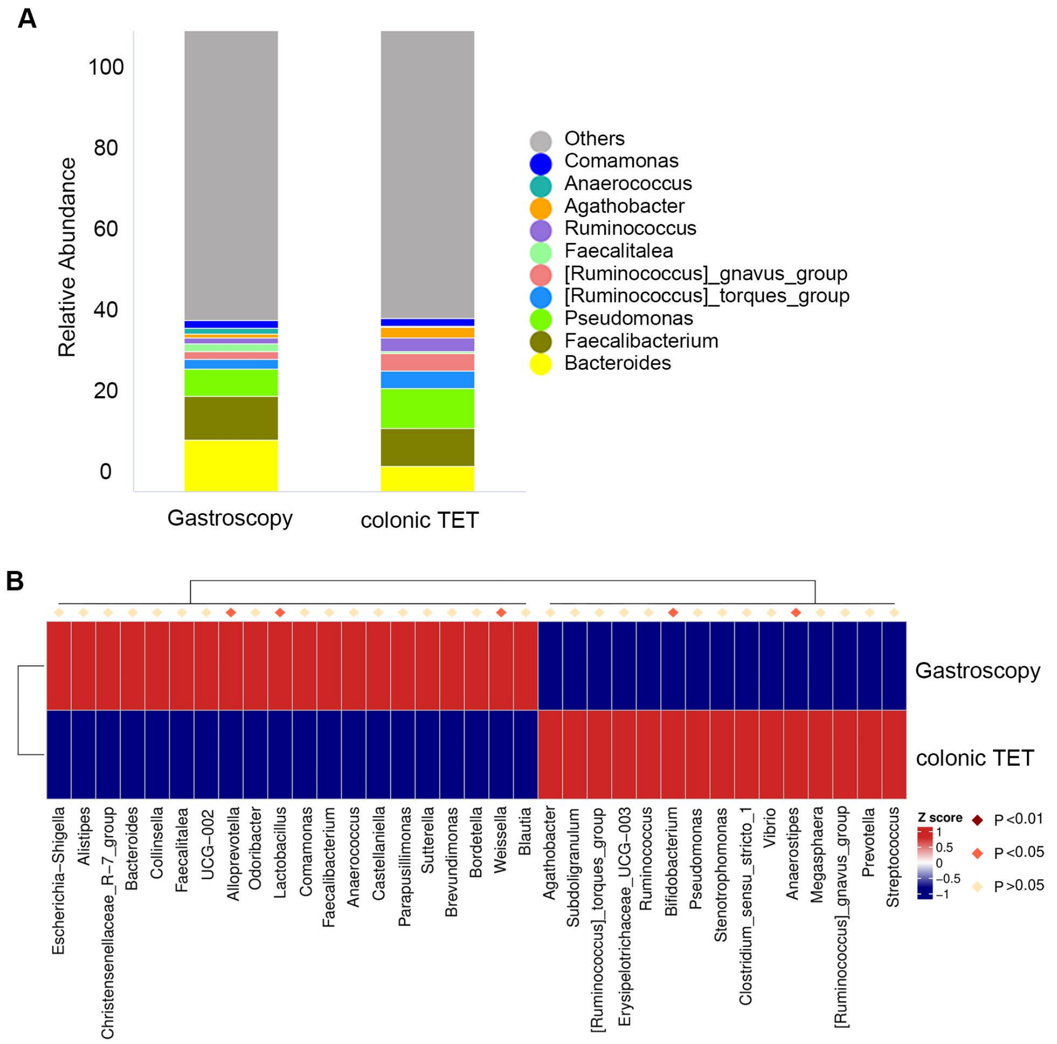

Supplement figure7

Percentage of Ig bound bacteria has no difference between pre/post FMT,delivery method,different donor

Supplementary Figure8

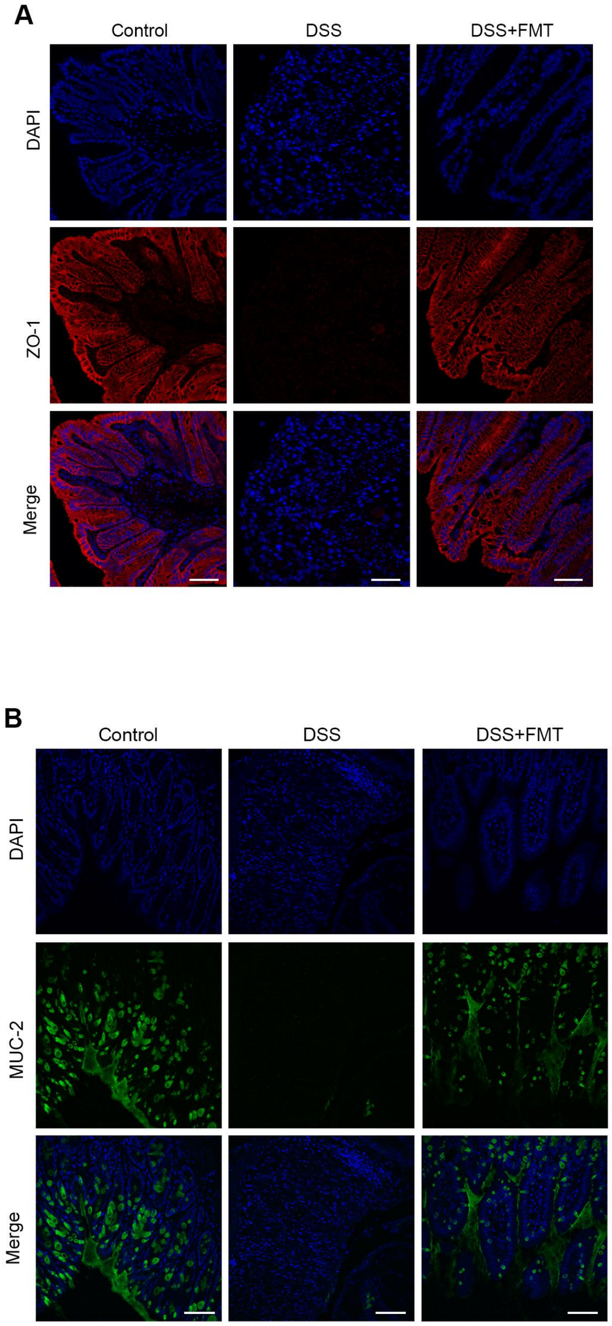

Supplement figure8

Immunofluorescence of ZO1 and muc2

Supplementary Figure9

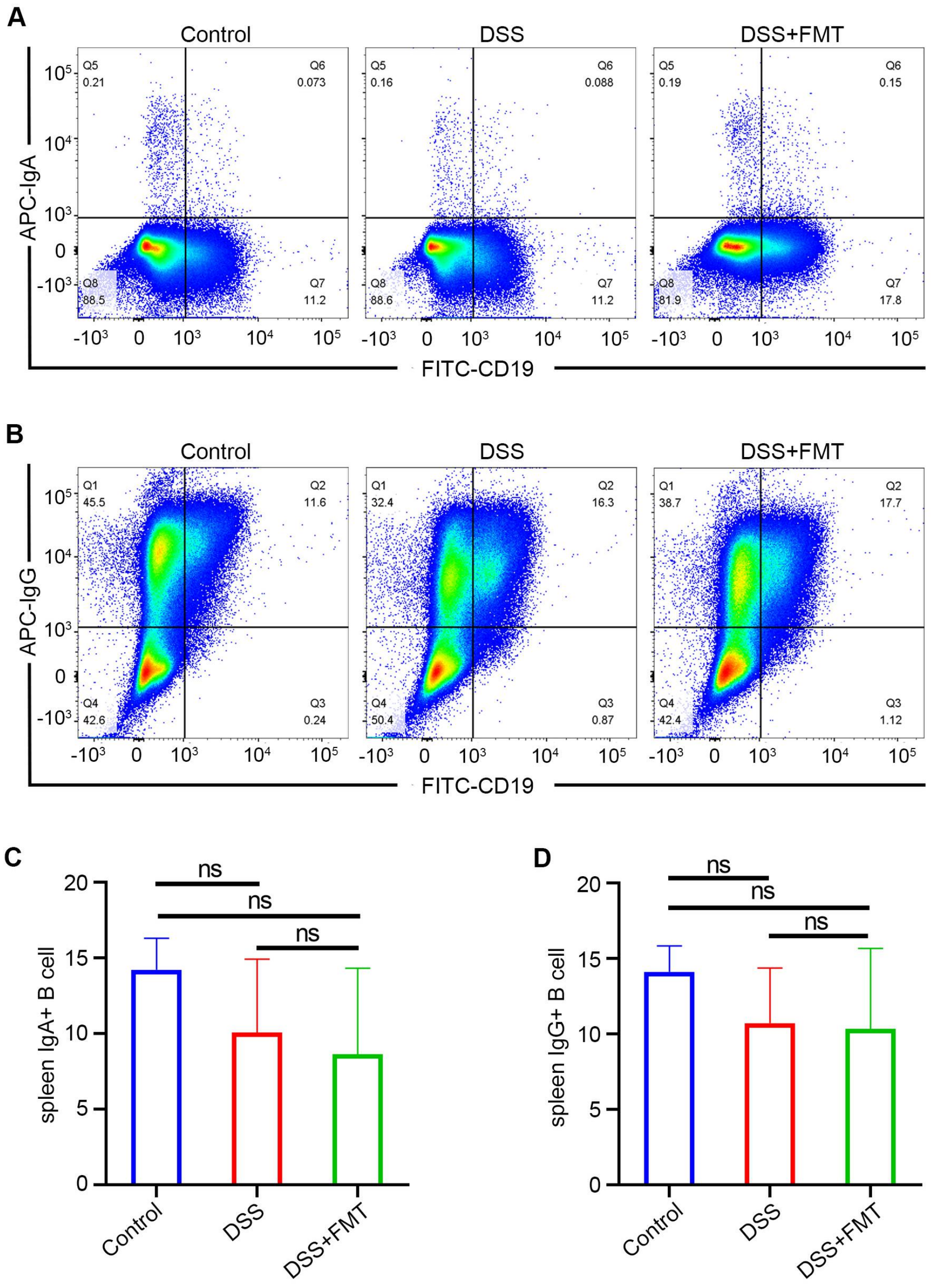

Supplement figure9

IgA/G+ B cells of spleen were determined by flow cytometry.

Supplementary Figure10

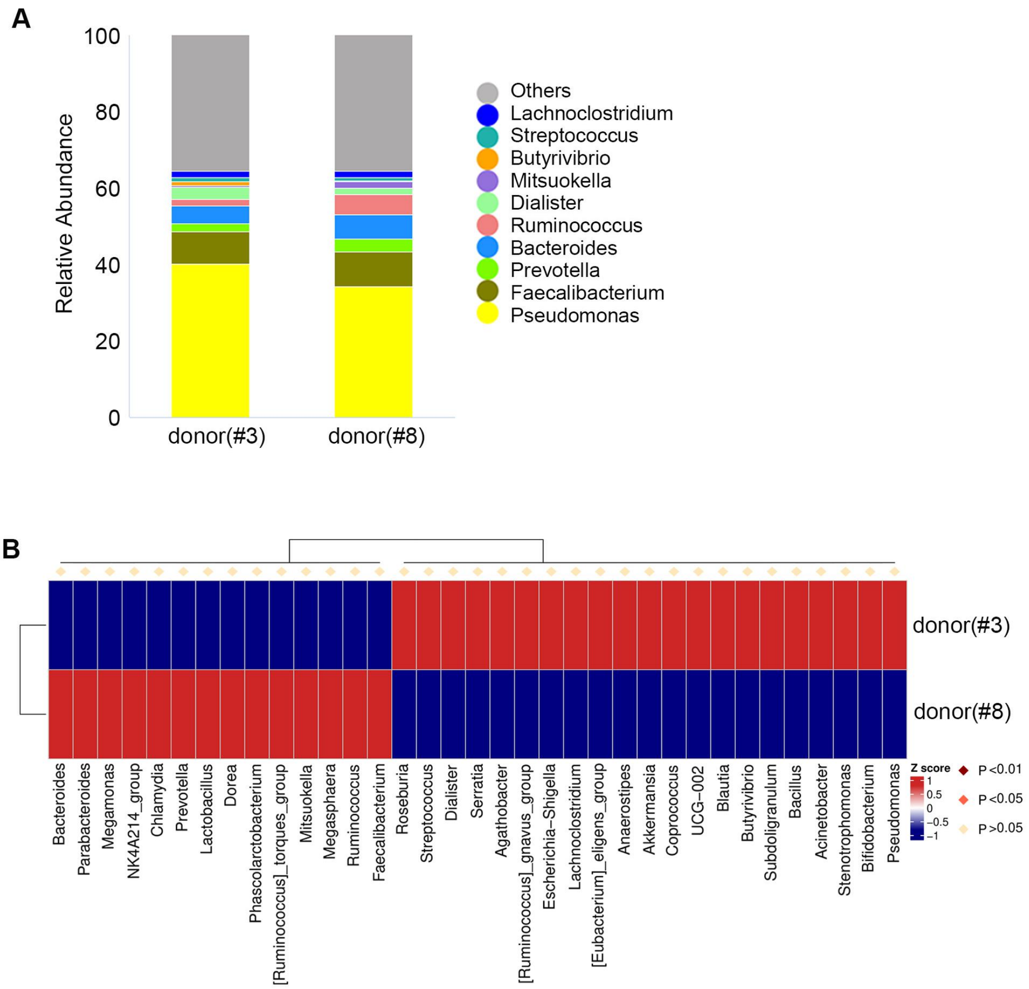

Supplement figure10

Characteristic of IgA bound bacteria of post FMT by different donor

A. Stacked bar plot of genera

B. Heatmap analysis of the difference in genera
